# Colostrum Quality as an Indicator of the Immune Status of Cows and Its Association with Peripartum Disease Risk in a Grazing Dairy Herd

**DOI:** 10.3390/ani15070958

**Published:** 2025-03-27

**Authors:** Maria Jaureguiberry, Santiago G. Corva, Taiel P. Konis, Maria J. Marconi, Ana L. Migliorisi, Maria G. Salas, German A. Dominguez, R. Luzbel de la Sota, Mauricio J. Giuliodori, Laura V. Madoz

**Affiliations:** 1 Instituto de Investigaciones en Reproducción Animal (INIRA), Facultad de Ciencias Veterinarias (FCV), Universidad Nacional de la Plata (UNLP), La Plata 1900, Argentina; mjaureguiberry@fcv.unlp.edu.ar (M.J.); tpkonis@fcv.unlp.edu.ar (T.P.K.); mjmarconi@fcv.unlp.edu.ar (M.J.M.); lmiglio@fcv.unlp.edu.ar (A.L.M.); msalas@fcv.unlp.edu.ar (M.G.S.); luzbel@fcv.unlp.edu.ar (R.L.d.l.S.); 2 Consejo Nacional de Investigaciones Científicas y Técnicas (CONICET), CABA 1425, Argentina; 3 Cátedra de Epidemiología y Salud Pública, FCV-UNLP, La Plata 1900, Argentina; sgcorva@isis.unlp.edu.ar; 4 Private Practice, Santa Fe, Venado Tuerto 2600, Argentina; germandominguez@powervt.com.ar; 5 Cátedra de Fisiología, FCV-UNLP, La Plata 1900, Argentina

**Keywords:** immunity, Brix score, periparturient disease risk, dairy cow

## Abstract

This study aimed to evaluate the association between colostrum quality, as indicative of the immune status of the cows, and peripartum disease risk occurrence in a herd of grazing dairy cows. A prospective cohort study was conducted on a grazing dairy farm with 3000 Holstein milking cows from 15 March 2022 to 15 March 2023. Multivariable binary logistic models evaluated the association between colostrum quality (expressed in Brix grades as a continuous predictor) and the risk of peripartum diseases (including dystocia, stillbirth, retention of fetal membranes, metritis, and clinical endometritis). We found that colostrum quality was negatively associated with the risk of dystocia (*p* = 0.02), stillbirth (*p* < 0.01), clinical endometritis (*p* = 0.02), and total peripartum diseases (*p* < 0.01). Conversely, colostrum quality was not related to the risk of retention of the placenta (*p* = 0.25) or metritis (*p* = 0.76). Additionally, we found that the calving season affected colostrum quality (*p* < 0.01). Conversely, parity number, dry period length, and milk yield in previous lactation did not affect it (*p* > 0.10). We concluded that colostrum quality, expressed in Brix grades, is negatively associated with peripartum disease risk in dairy cows and could be a useful indicator of the immune status of the cow.

## Simple Summary

The periparturient period is crucial for cows as they prepare to give birth and produce milk. This period is challenging because cows experience negative energy balance, hypocalcemia, oxidative stress, systemic inflammation, and a weakened immune response, leading them to an increased risk of peripartum diseases. Of all the above challenges, the one that has received the least attention from the scientific community is the immune status of cows. Colostrum quality evaluation is a common on-farm practice to measure the source of immunity for newborn calves. We believe that colostrum quality can also be an indicator of the immune status of the cows producing it. Therefore, we conducted a study to evaluate the association between colostrum quality, as a potential indicator of the immune status of the cows, and the risk of developing peripartum diseases in a herd of grazing dairy cows. We found that cows with higher colostrum quality had lower risks of diseases, such as stillborn, dystocia, and endometritis. Our findings suggest that colostrum quality evaluation could be a helpful tool not only for the potential transfer of immunity to calves but also for the indirect assessment of the immune status of cows.

## 1. Introduction

The periparturient or transition period, 21 d before to 21 d after parturition, is characterized by several changes in the endocrine and immune systems, where cows prepare for colostrum secretion, parturition, and milk production [1,2,3]. This critical transition from a non-lactating pregnant state to a lactating non-pregnant state is highly demanding [1,2,4,5]. In this period, the cows face many challenges, such as negative energy balance [1,2,3], hypocalcemia [4,6], oxidative stress [7,8,9], and systemic inflammation [8,9,10,11]. At the same time, this transition period is also characterized by a reduction in immune competence [4,12,13,14,15]. That is why most diseases occur during parturition and the first weeks of lactation [16,17,18,19]. There is consensus that diseases harm productivity, given that diseased dairy cows may experience a reduction in milk yield, are less likely to become pregnant, and are more likely to be culled [16,17,20]. Postpartum diseases (e.g., metritis) cause economic losses due to the cost of treatment, decreased milk production, lower reproductive efficiency, and increased mortality rate [21,22] and impair animal welfare [23]. It is well known that pathogens invade the uterus at parturition, increasing the risk of developing uterine infections like metritis [24]. So, it is key for cows to build a robust immune response to eliminate potentially harmful bacteria from their uterus after calving [25]. Cows that fail to do that are more likely to develop metritis and endometritis and require antimicrobial therapy [21,26]. There is a growing concern under the One Health paradigm that the overuse and misuse of antimicrobials have increased bacterial resistance [27,28]. Therefore, reducing the number of affected cows would help to diminish the use of antimicrobials [29].

Regarding transition period management, various ways of evaluating the level of negative energy balance (e.g., the blood concentration of free fatty acid and BHBA), hypocalcemia (e.g., blood Ca concentration), oxidative stress (e.g., reactive oxygen species and total antioxidant capacity), and overt inflammatory response (e.g., blood concentration of acute phase proteins) have been proposed [7,11,30,31]. However, there is no simple method to assess the immune status of cows. In this sense, colostrum quality evaluation could be used to estimate the immune status of the cow and, thus, predict the occurrence of peripartum disease simply and quickly. As is known, colostrum is a complex secretion that is produced by the mammary gland several weeks before calving. Its principal components are proteins, especially immunoglobulins, leukocytes, hormones, growth factors, and nutrients [32]. Generally, its quality is tested in dairy farms to provide good colostrum to calves after parturition as soon as possible. This management practice plays a key role in the success of the calf-rearing period. The most effective, practical, and economical on-farm tool commonly used for measuring colostrum quality is the Brix refractometer. It does so indirectly by measuring the total solid concentration [33]. A Brix score of 22% or higher is usually recommended as a threshold to determine good-quality colostrum for calves [32]. To our knowledge, immune status at parturition, measured by colostrum quality, has not been evaluated as a predictor of disease occurrence in dairy cows. Therefore, we propose the use of colostrum quality as an indicator of the immune status of the cows that produce it instead of using it exclusively as a source of immunity for calves.

To test the proposed hypothesis that the immune status of the cows is associated with the disease risk of grazing dairy cows, we conducted a prospective cohort study to evaluate the association between colostrum quality, as indicative of the immune status of the cows, and the chance of peripartum diseases occurrence in a herd of grazing dairy cows.

## 2. Materials and Methods

### 2.1. Study Farm

This study was performed on a commercial dairy farm in Carlos Casares (35°37′ S, 61°22′ W), Buenos Aires province, Argentina, with 3000 milking cows. The herd’s average milk production was around 10,000 kg. Breeding occurred year-round except for the hot Summer months (January and February). Cows approaching their expected delivery date were placed in dry lots and fed a diet with a low cation–anion difference. Trained farm staff closely monitored the cows for signs of delivery. Permanent veterinary staff were available to assist the cows if necessary. Cows that experienced difficulties during parturition and required assistance were classified as having dystocia. Stillbirth was defined as the death of a calf that occurs after at least 260 days of gestation just before, during, or within 24 to 48 h after parturition, while retention of fetal membranes was considered when a cow had not expelled the membranes within 24 h after calving. After parturition, a colostrum sample was collected, and the Brix score was measured using a clinical hand-held and automatic temperature compensation refractometer following the manufacturer’s instructions (Alla France, Chemillé en Anjou, France).

All cows were checked using a Metricheck (Simcro, Hamilton, New Zealand) device for vaginal discharge at 7 ± 3 days postpartum (DPP). Vaginal discharge was classified using a 0–3 scale: VD0 (normal clear discharge), VD1 (clear discharge with pus flecks), VD2 (mucopurulent, not fetid discharge), and VD3 (watery, purulent, or brown-colored and fetid). Cows with VD1-3 were rechecked at 14 ± 3 and 21 ± 3 DPP. Cows with VD0 were considered healthy and were assumed to keep VD0 without requiring further checks during the voluntary waiting period. Cows presenting a VD3 during the first 21 DPP were considered presenting metritis and were treated with a single subcutaneous administration of 6.6 mg/kg ceftiofur crystalline-free acid (Excede sterile suspension; Zoetis, Martinez, Argentina). All diagnosed cows were re-examined with Metricheck weekly to confirm case remission. Cows presenting VD1-2 were left untreated. Finally, cows having fetid vaginal discharge (VD3) after 21 DPP and until the end of the waiting voluntary period (45 DPP) were diagnosed with clinical endometritis (CE). All cows diagnosed with CE were treated once with an intrauterine suspension of 500 mg of cephapirin benzathine (Metricure^®^; MSD, Munro, Argentina).

### 2.2. Dataset Management

This prospective cohort study included all the cows calving from 15 March 2022 to 15 March 2023. Data were extracted from commercial software (Protambo Master 3.5; DIRSA S.A., Gonnet, Argentina). Data about the dry period length, calving condition, colostrum quality (in Brix scores), clinical and reproductive examinations, treatments applied, reproductive management, and milk production were recorded. Seasons of parturition were considered as follows: Summer (21 December to 20 March), Fall (21 March to 20 June), Winter (21 June to 20 September), and Spring (21 September to 20 December). Criteria for cow record inclusion were having information about colostrum quality (in Brix scores), calving condition, dry period length, a complete history of vaginal discharge, postpartum disease diagnosis, and the treatments applied.

### 2.3. Statistical Analysis

Descriptive data (e.g., colostrum quality) were obtained using the Proc Univariate of SAS. Univariable linear models were used to assess the effect of calving season (Summer vs. Fall vs. Winter vs. Spring), parity number (primiparous vs. multiparous), dry period length (≤45 vs. 46–64 vs. ≥65 days), and milk yield in previous lactation (<7000 vs. ≥7000 to <9000 vs. ≥9000 kg) on colostrum quality (in Brix scores) with Proc Glimmix of SAS using normal distribution and identity link function. Then, a final multivariable linear model was offered with predictors having a *p* < 0.10. Those predictors remain if their *p* < 0.10, except colostrum quality, which was forced to remain. Univariable logistic models were run to assess the association between colostrum quality (in Brix scores as a continuous predictor), the same predictors described for the linear model (calving season, parity, dry period length, and previous lactation milk yield), and the risk of peripartum diseases (such as dystocia, retention of the placenta, stillbirth, metritis, and endometritis); these were models were created with Proc Glimmix of SAS using binomial distribution and logit link function. Then, final multivariable logistic models were offered with predictors having a *p* < 0.10, where they remain if *p* < 0.10, except for colostrum quality, which was forced to remain. The odds for diseases were evaluated with different models: 1—dystocia (no vs. yes), 2—retention of the placenta (no vs. yes), 3—stillbirth (no vs. yes), 4—parturition diseases (all the above, no vs. yes), 2—metritis (no vs. yes), 3—endometritis (no vs. yes), and 4—all diseases together (healthy vs. diseased). As colostrum quality was included as a continuous predictor, its odds are expressed per one unit of increment in Brix scores over the mean. According to Allison [34], it is helpful to convert the odds ratio into percentages for quantitative predictors with the following formula: odds ratio minus 1 multiplied by 100 (% = OR − 1 × 100). Therefore, this conversion gave the percent change in the odds per one unit of increment in the quantitative predictor. Statistical significance was set at *p* < 0.05, and a tendency was set at *p* ≤ 0.10. All analyses were conducted in SAS^®^ On Demand for Academics 3.81 Enterprise Edition (SAS Institute Inc., 27513-2414 Cary, NC, USA).

## 3. Results

### 3.1. Descriptive Analysis

The records of 2770 cows were included in the study. In total, 32% (n: 883) were primiparous, 28% (n: 774) second-parity, 24% (n: 662) third-parity, and 16% (n: 451) fourth-parity or higher. The average milk yield in the previous lactation was 9657 ± 2699 (mean ± SD). In total, 13% of cows (n: 247) had a prior lactation production of ≤7000 kg, 31% of cows (n: 589) produced between 7001 and 9000 kg, and the remaining 56% of cows (n: 1047) produced > 9000 kg. The distribution of parturitions by season during the study period was 12% (320/2770) in Summer, 34% (937/2770) in Autumn, 31% (855/2770) in Winter, and 24% (658/2770) in Spring. In total, 1862 cows had complete data about the dry period length. In total, 2% (41/1862) of cows had a dry period of ≤45 d, 35% (651/1862) had 46–64 d, and 63% of cows (1170/1862) had ≥65 d. The mean ± SD colostrum quality in Brix scores was 25 ± 2%.

### 3.2. Colostrum Quality

A linear model showed that calving season (*p* < 0.01) affected colostrum quality, given that quality was highest in Fall and Spring, intermediate in Winter, and lowest in Summer (Table 1). Conversely, parity, dry period length, and milk yield in previous lactation did not affect colostrum quality (*p* > 0.10, Table 1).

### 3.3. Disease Risk

Logistic models showed that colostrum quality affects the risk of dystocia (OR = 0.86, *p* = 0.02), stillbirth (OR = 0.64, *p* < 0.01), parturition disease (OR = 0.79, *p* < 0.01), clinical endometritis (OR = 0.93, *p* = 0.02), and total diseases (OR = 0.93, *p* = 0.01, Table 2). Conversely, colostrum quality was not associated with the risk of retention of the placenta (*p* = 0.25) and metritis (*p* = 0.76, Table 2). Calving season affected the risk of dystocia (*p* = 0.03), stillbirth (*p* = 0.01), parturition disease (*p* < 0.01), metritis (*p* = 0.01), clinical endometritis (*p* = 0.04), and total diseases (*p* < 0.01, Table 3). Parity tended to affect the risk of placental retention (*p* = 0.09) and the risk of total diseases (*p* = 0.01, Table 3). The dry period length was related to the risk of placental retention (*p* < 0.01) and tended to affect the risk of stillbirth (*p* = 0.09, Table 3). Finally, milk yield in previous lactation tended to affect the risk of metritis (*p* = 0.07) and affected the risk of total diseases (*p* < 0.01, Table 3).

## 4. Discussion

Colostrum quality, expressed in Brix scores, was negatively associated with the risk of dystocia, stillbirth, clinical endometritis, parturition diseases, and total diseases in a herd of grazing dairy cows (*p* < 0.05). In this sense, the risk of dystocia, stillbirth, parturition diseases (dystocia, stillbirth, and retention of fetal membranes), endometritis, and total diseases decreased, respectively, by 14%, 36%, 21%, 7%, and 7% per every increase in % of Brix scores over the mean. This supports the proposed hypothesis that the immune status of cows is associated with the risk of disease and that colostrum quality could be used as an indicator of the immune status of the cows.

The findings align with those of Stoop et al. [35], who found that high immune responding dairy cows have fewer economically significant diseases, such as mastitis, metritis, ketosis, and retained placenta. Stoop et al. [35] also found that these high immune responders with improved disease resistance produce more antibodies following vaccination and have a better colostrum quality. In addition, studies in housed North American dairies showed that Holstein Friesian cows with enhanced general immune responsiveness have a reduced incidence of diseases [36,37]. In this sense, Fleming et al. [38] showed that Canadian Holstein Friesian cows with enhanced antibody and cell-mediated immune responsiveness produce more colostral immunoglobulins than low-responding herd-mates. Considering that reported heritability estimates are 0.32 and 0.64 for humoral and cell immune response, it would be feasible to include immune traits in breeding programs [37,39]. Therefore, selecting dairy cows for improved disease resistance would have the potential to help cows better adapt to and manage the challenges imposed by their production environment [40,41]. There is consensus that one of the biggest challenges dairy cows will face is reaching a high milk yield while maintaining their health and welfare. Thus, there is a need to develop new technologies that enable the early detection of problems in milk production, health, or animal welfare so that action can be taken as soon as possible to ameliorate harmful effects [42]. Therefore, colostrum quality evaluation (e.g., Brix scores) could be used as a biomarker of the immune/health status of the cows.

The colostrum quality found (25% Brix scores) was similar to previous reports, such as 26% [33] and 24% [43,44]. The finding that the calving season affects colostrum quality (e.g., quality is lowest in Summer) agrees with a previous report [33]. A similar effect was observed for cold stress in Winter-calving cows in Norway [45]. A possible explanation could be that heat stress (in Summer) reduces dry matter intake and mammary blood flow, decreasing the transfer of immunoglobulins (and nutrients) to the udder [32].

Conversely, we found no effect of parity number, dry period length, and previous lactation yield on colostrum quality. Some reports have shown that second-parity cows have the lowest total solids and IgG content [33,45]. However, other studies showed that Ig concentration in colostrum is correlated with the number of lactations, with levels increasing from the first parturition onwards [46,47]. It has been proposed that older cows, which have had a more prolonged exposure to antigens, produce colostrum with higher levels of antibodies [45,48].

Regarding the dry period length, the findings align with previous reports [33,49]. Soufleri et al. [33] found no significant influence of dry period length on the total solid content of colostrum. Similarly, Westhoff et al. [49] found that shortened (not omitted) dry periods reduced the colostrum yield but did not affect its quality. O’Hara et al. [50] showed that cows with a one-month dry period produced less colostrum but had a higher protein content than those with longer periods.

The finding that previous lactation milk yield did not affect colostrum quality disagrees with a report showing that preceding lactation milk yield and colostrum’s Ig concentration have a mild positive correlation [48].

Regarding the strengths of the present work, it is the first study assessing the association of colostrum quality, as an indicator of the immune status of cows at parturition, with the risk of peripartum diseases in dairy cows. Previous studies have exclusively evaluated colostrum quality as calves’ immunity source [32]. This new approach enabled us to obtain promising results about extending its use for the practical evaluation of the immune status of cows at the farm level. Regarding the limitations, given that the study design involves only one big dairy farm, it is impossible to account for the farm effect on the relationship between colostrum quality and disease risk. In this sense, a study by Gulliksen et al. [45] involving 119 herds showed high variability in Ig content in colostrum between herds. In addition, as colostrum quantity was not recorded in the present study, it is impossible to evaluate if the volume of colostrum produced would have any dilutional effect, which was found by Godden et al. [32], who stated that cows producing more than 8.5 kg of colostrum have lower quality than lower-producing cows, and Westhoff et al. [49], who reported that lower colostrum yield increased colostrum quality. Finally, as the present study did not measure Ig concentration in colostrum or blood samples, the immune status of the cows was indirectly assessed through colostrum quality evaluation (in Brix scores).

## 5. Conclusions

Colostrum quality, expressed in Brix scores, is negatively associated with parturition and postpartum uterine disease risk (e.g., dystocia, dead calf, and endometritis) in dairy cows. These findings suggest that colostrum quality evaluation could be used not only for the potential transfer of immunity to calves but also for the indirect assessment of the immune status of cows.

## Figures and Tables

**Table 1:** Linear model assessing the effect of calving season, parity number, dry period length, and total milk yield in previous lactation on colostrum quality (in Brix scores [%]) in a herd of highly supplemented grazing dairy cows (n = 2770).

		Brix Scores (Mean ± SE)	SE	*p*
Calving season				<0.01
	Fall	25.68 ª	0.05	
	Winter	25.32 ^b^	0.05	
	Spring	25.69 ª	0.06	
	Summer	25.03 ^c^	0.08	
Parity number				0.33
	1	25.55	0.05	
	2	25.54	0.06	
	3+	25.50	0.05	
Dry period length (d)				0.39
	<45	25.51	0.05	
	45–64	25.52	0.04	
	≥65	25.50	0.05	
Previous lactation milk yield (kg)				0.43
	<7000	25.54	0.05	
	7000–9000	25.48	0.06	
	>9000	25.46	0.05	

**Table 2:** Logistic model assessing the effect of colostrum quality (in Brix scores [%] as a continuous predictor) on the risk of parturition and postpartum uterine diseases adjusting for calving season, parity number, and dry period length in a herd of highly supplemented grazing dairy cows (n = 2770).

Effect	^1^ OR	^2^ 95% CI	*p*
^3^ Colostrum quality (Brix scores)	^4^ Risk of retention of fetal membranes		
	1.22	0.87–1.75	0.25
	^5^ Risk of dystocia		
	0.86	0.77–0.97	0.02
	^6^ Risk of stillbirth		
	0.64	0.56–0.73	<0.01
	^7^ Risk of parturition diseases		
	0.79	0.72–0.87	<0.01
	^8^ Risk of metritis		
	0.99	0.92–1.06	0.76
	^9^ Risk of clinical endometritis		
	0.93	0.87–0.99	0.02
	^10^ Risk of diseases		
	0.93	0.88–0.98	0.01

^1^ OR: odds ratio expressed per one unit of increment in Brix scores over the mean (25%); ^2^ 95% CI: confidence interval of the odds ratio; ^3^ Colostrum quality: in Brix scores; ^4^ Retention of fetal membranes: cows that have not expelled fetal membranes within 24 h after calving; ^5^ Dystocia: cows with calving difficulties that need assistance during parturition; ^6^ Stillbirth: cows experiencing the death of a calf that occurs after at least 260 days of gestation just before, during, or within 24 to 48 h after parturition; ^7^ Parturition diseases: cows having retention of fetal membranes, dystocia, or stillbirths; ^8^ Metritis: cows having watery, purulent, or brown-colored and fetid vaginal discharge during the first 21 days postpartum (DPP); ^9^ Clinical endometritis: cows having watery, purulent, or brown-colored and fetid vaginal discharge after 21 DPP; ^10^ Diseases: cows having any of the above diseases.

**Table 3:** Logistic model assessing the effect of calving season, parity number, previous lactation milk yield, and dry period length on the risk of parturition and postpartum uterine diseases in a herd of highly supplemented grazing dairy cows (n = 2770).

Effect	Levels	^1^ %	^2^ OR	^3^ 95% CI	*p*
		^4^ Risk of retention of fetal membranes			
Parity	≥3	<0.1	Ref.		0.09
	2	<0.1	0.17	0.02–1.32	
	1	7	^5^ NE		
Dry period (d)	45–64	0.4	Ref.		<0.01
	<45	<0.1	^5^ NE		
	≥65	0.4	1.15	0.29–4.48	
		^6^ Risk of dystocia			
Calving season	Winter	3	Ref.		0.03
	Fall	2	0.95	0.57–1.71	
	Spring	5	1.95	1.13–3.38	
	Summer	3	1.36	0.67–2.77	
		^7^ Risk of stillbirth			
Calving season	Winter	1	Ref.		0.01
	Fall	2	1.36	0.67–2.75	
	Spring	4	2.75	1.40–5.38	
	Summer	1	1.01	0.38–2.68	
Dry period (d)	45–64	2	Ref.		0.09
	<45	3	2.27	0.75–6.94	
	≥65	1	0.70	0.37–1.31	
		^8^ Risk of parturition diseases			
Calving season	Winter	4	Ref.		<0.01
	Fall	4	0.99	0.62–1.58	
	Spring	9	2.42	1.57–3.73	
	Summer	4	0.99	0.52–1.86	
		^9^ Risk of metritis			
Calving season	Winter	13	Ref.		0.01
	Fall	15	1.15	0.88–1.51	
	Spring	16	1.27	0.95–1.70	
	Summer	8	0.60	0.38–0.93	
Previous lactation milk yield (kg)	<7000	12	Ref.		0.07
	7000–9000	12	1.00	0.74–1.35	
	>9000	15	1.30	1.02–1.66	
		^10^ Risk of clinical endometritis			
Calving season	Winter	14	Ref.		0.04
	Fall	17	1.27	0.98–1.64	
	Spring	15	1.07	0.80–1.44	
	Summer	11	0.74	0.50–1.11	
		^11^ Risk of total diseases			
Calving season	Winter	25	Ref.		<0.01
	Fall	27	1.19	0.96–1.48	
	Spring	29	1.31	1.04–1.65	
	Summer	17	0.67	0.48–0.93	
Parity	≥3	21	Ref.		0.01
	2	21	1.01	0.82–1.25	
	1	31	1.74	1.17–2.54	
Previous lactation milk yield (kg)	<7000	20	Ref.		0.03
	7000–9000	23	1.20	0.82–1.75	
	>9000	30	1.68	1.18–2.39	

^1^ %: mean values obtained with LSM option; ^2^ OR: odds ratio; ^3^ 95% CI: confidence interval of the odds ratio; ^4^ Retention of fetal membranes: cows that have not expelled fetal membranes within 24 h after calving; ^5^ NE: non estimated; ^6^ Dystocia: cows with calving difficulties that need assistance during parturition; ^7^ Stillbirth: cows experiencing the death of a calf that occurs after at least 260 days of gestation just before, during, or within 24 to 48 h after parturition; ^8^ Parturition diseases: cows having retention of fetal membranes, dystocia, or stillbirths; ^9^ Metritis: cows having watery, purulent, or brown-colored and fetid vaginal discharge during the first 21 days postpartum (DPP); ^10^ Clinical endometritis: cows having watery, purulent, or brown-colored and fetid vaginal discharge after 21 DPP; ^11^ Total diseases: cows having any of the above diseases. Predictors with a *p* ≥ 0.10 were not shown.

## Data Availability

The original contributions presented in this study are included in the article. Further inquiries can be addressed to the corresponding author.

## References

[1] Drackley J.K. (1999). Biology of Dairy Cows During the Transition Period: The Final Frontier?. J. Dairy Sci..

[2] Grummer R.R. (1995). Impact of Changes in Organic Nutrient Metabolism on Feeding the Transition Dairy Cow. J. Anim. Sci..

[3] Grummer R.R. (1993). Etiology of Lipid-Related Metabolic Disorders in Periparturient Dairy Cows. J. Dairy Sci..

[4] Goff J.P., Horst R.L. (1997). Physiological Changes at Parturition and Their Relationship to Metabolic Disorders. J. Dairy Sci..

[5] Van Knegsel A.T.M., Hammon H.M., Bernabucci U., Bertoni G., Bruckmaier R.M., Goselink R.M.A., Gross J.J., Kuhla B., Metges C.C., Parmentier H.K. (2014). Metabolic Adaptation during Early Lactation: Key to Cow Health, Longevity and a Sustainable Dairy Production Chain. CABI Rev. Perspect. Agric. Vet. Sci. Nutr. Nat. Resour..

[6] DeGaris P.J., Lean I.J. (2008). Milk Fever in Dairy Cows: A Review of Pathophysiology and Control Principles. Vet. J..

[7] Bernabucci U., Ronchi B., Lacetera N., Nardone A. (2005). Influence of Body Condition Score on Relationships between Metabolic Status and Oxidative Stress in Periparturient Dairy Cows. J. Dairy Sci..

[8] Bionaz M., Trevisi E., Calamari L., Librandi F., Ferrari A., Bertoni G. (2007). Plasma Paraoxonase, Health, Inflammatory Conditions, and Liver Function in Transition Dairy Cows. J. Dairy Sci..

[9] Sordillo L.M., Contreras G.A., Aitken S.L. (2009). Metabolic Factors Affecting the Inflammatory Response of Periparturient Dairy Cows. Anim. Health Res. Rev. Conf. Res. Work. Anim. Dis..

[10] Bertoni G., Trevisi E. (2013). Use of the Liver Activity Index and Other Metabolic Variables in the Assessment of Metabolic Health in Dairy Herds. Vet. Clin. N. Am.—Food Anim. Pract..

[11] Trevisi E., Amadori M., Cogrossi S., Razzuoli E., Bertoni G. (2012). Metabolic Stress and Inflammatory Response in High-Yielding, Periparturient Dairy Cows. Res. Vet. Sci..

[12] Kehrli M.E., Nonnecke B.J., Roth J.A. (1989). Alterations in Bovine Neutrophil Function during the Periparturient Period. Am. J. Vet. Res..

[13] Kehrli M.E., Nonnecke B.J., Roth J.A. (1989). Alterations in Bovine Lymphocyte Function during the Periparturient Period. Am. J. Vet. Res..

[14] Lacetera N., Scalia D., Bernabucci U., Ronchi B., Pirazzi D., Nardone A. (2005). Lymphocyte Functions in Overconditioned Cows around Parturition. J. Dairy Sci..

[15] Mallard B.A., Dekkers J.C., Ireland M.J., Leslie K.E., Sharif S., Vankampen C.L., Wagter L., Wilkie B.N. (1998). Alteration in Immune Responsiveness during the Peripartum Period and Its Ramification on Dairy Cow and Calf Health. J. Dairy Sci..

[16] Carvalho M.R., Peñagaricano F., Santos J.E.P., DeVries T.J., McBride B.W., Ribeiro E.S. (2019). Long-Term Effects of Postpartum Clinical Disease on Milk Production, Reproduction, and Culling of Dairy Cows. J. Dairy Sci..

[17] Dubuc J., Duffield T.F., Leslie K.E., Walton J.S., LeBlanc S.J. (2011). Effects of Postpartum Uterine Diseases on Milk Production and Culling in Dairy Cows. J. Dairy Sci..

[18] Hostens M., Ehrlich J., Van Ranst B., Opsomer G. (2012). On-Farm Evaluation of the Effect of Metabolic Diseases on the Shape of the Lactation Curve in Dairy Cows through the MilkBot Lactation Model. J. Dairy Sci..

[19] Santos J.E., Cerri R.L., Ballou M.A., Higginbotham G.E., Kirk J.H. (2004). Effect of Timing of First Clinical Mastitis Occurrence on Lactational and Reproductive Performance of Holstein Dairy Cows. Anim. Reprod. Sci..

[20] Chiozza Logroño J., Rearte R., Corva S.G., Domínguez G.A., de la Sota R.L., Madoz L.V., Giuliodori M.J. (2021). Lameness in Early Lactation Is Associated with Lower Productive and Reproductive Performance in a Herd of Supplemented Grazing Dairy Cows. Animals.

[21] Giuliodori M.J., Magnasco R.P., Becu-Villalobos D., Lacau-Mengido I.M., Risco C.A., De la Sota R.L. (2013). Metritis in Dairy Cows: Risk Factors and Reproductive Performance. J. Dairy Sci..

[22] de la Sota R.L., Corva S., Dominguez G., Madoz L.V., Jaureguiberry M., Giuliodori M. (2020). Analysis of Puerperal Metritis Treatment Records in a Grazing Dairy Farm in Argentina. Tierarztl. Prax. Ausg. G Grosstiere—Nutztiere.

[23] LeBlanc S.J. (2023). Review: Postpartum Reproductive Disease and Fertility in Dairy Cows. Animal.

[24] Hammon D.S., Evjen I.M., Dhiman T.R., Goff J.P., Walters J.L. (2006). Neutrophil Function and Energy Status in Holstein Cows with Uterine Health Disorders. Vet. Immunol. Immunopathol..

[25] Machado V.S., Silva T.H. (2020). Adaptive Immunity in the Postpartum Uterus: Potential Use of Vaccines to Control Metritis. Theriogenology.

[26] Madoz L.V., Prunner I., Jaureguiberry M., Gelfert C.C., de la Sota R.L., Giuliodori M.J., Drillich M. (2017). Application of a Bacteriological On-Farm Test to Reduce Antimicrobial Usage in Dairy Cows with Purulent Vaginal Discharge. J. Dairy Sci..

[27] Tang K.W.K., Millar B.C., Moore J.E. (2023). Antimicrobial Resistance (AMR). Br. J. Biomed. Sci..

[28] Cuong N.V., Padungtod P., Thwaites G., Carrique-Mas J.J. (2018). Antimicrobial Usage in Animal Production: A Review of the Literature with a Focus on Low-and Middle-Income Countries. Antibiotics.

[29] Health for Animals (2024). How Prevention Can Reduce the Need for Antibiotics Pathways to Reduce the Need for Antimicrobials on Farms for Sustainable Agrifood Systems Transformation (RENOFARM). FAO Report.

[30] Ospina P.A., Nydam D.V., Stokol T., Overton T.R. (2010). Associations of Elevated Nonesterified Fatty Acids and β-Hydroxybutyrate Concentrations with Early Lactation Reproductive Performance and Milk Production in Transition Dairy Cattle in the Northeastern United States. J. Dairy Sci..

[31] Trevisi E., Minuti A. (2018). Assessment of the Innate Immune Response in the Periparturient Cow. Res. Vet. Sci..

[32] Godden S.M., Lombard J.E., Woolums A.R. (2019). Colostrum Management for Dairy Calves. Vet. Clin. N. Am.—Food Anim. Pract..

[33] Soufleri A., Banos G., Panousis N., Fletouris D., Arsenos G., Kougioumtzis A., Valergakis G.E. (2021). Evaluation of Factors Affecting Colostrum Quality and Quantity in Holstein Dairy Cattle. Animals.

[34] Allison P.D. (1999). Logistic Regression Using SAS: Theory and Application.

[35] Stoop C.L., Thompson-Crispi K.A., Cartwright S.L., Mallard B.A. (2016). Short Communication: Variation in Production Parameters among Canadian Holstein Cows Classified as High, Average, and Low Immune Responders. J. Dairy Sci..

[36] Thompson-Crispi K.A., Sewalem A., Miglior F., Mallard B.A. (2012). Genetic Parameters of Adaptive Immune Response Traits in Canadian Holsteins. J. Dairy Sci..

[37] Thompson-Crispi K.A., Hine B., Quinton M., Miglior F., Mallard B.A. (2012). Short Communication: Association of Disease Incidence and Adaptive Immune Response in Holstein Dairy Cows. J. Dairy Sci..

[38] Fleming K., Thompson-Crispi K.A., Hodgins D.C., Miglior F., Corredig M., Mallard B.A. (2016). Short Communication: Variation of Total Immunoglobulin G and β-Lactoglobulin Concentrations in Colostrum and Milk from Canadian Holsteins Classified as High, Average, or Low Immune Responders. J. Dairy Sci..

[39] Wagter L.C., Mallard B.A., Wilkie B.N., Leslie K.E., Boettche P.J., Dekkers J.C.M. (2000). A Quantitative Approach to Classifying Holstein Cows Based on Antibody Responsiveness and Its Relationship to Peripartum Mastitis Occurrence. J. Dairy Sci..

[40] Aleri J.W., Hine B.C., Pyman M.F., Mansell P.D., Wales W.J., Mallard B., Fisher A.D. (2016). Periparturient Immunosuppression and Strategies to Improve Dairy Cow Health during the Periparturient Period. Res. Vet. Sci..

[41] Aleri J.W., Hine B.C., Pyman M.F., Mansell P.D., Wales W.J., Mallard B., Fisher A.D. (2015). Assessing Adaptive Immune Response Phenotypes in Australian Holstein-Friesian Heifers in a Pasture-Based Production System. J. Anim. Sci..

[42] Gabai G., Amadori M., Knight C.H., Werling D. (2018). The immune system is part of a whole-organism regulatory network. Res. Vet. Sci..

[43] Quigley J.D., Lago A., Chapman C., Erickson P., Polo J. (2013). Evaluation of the Brix Refractometer to Estimate Immunoglobulin G Concentration in Bovine Colostrum. J. Dairy Sci..

[44] Bartier A.L., Windeyer M.C., Doepel L. (2015). Evaluation of On-Farm Tools for Colostrum Quality Measurement. J. Dairy Sci..

[45] Gulliksen S.M., Lie K.I., Sølverød L., Østerås O. (2008). Risk Factors Associated with Colostrum Quality in Norwegian Dairy Cows. J. Dairy Sci..

[46] Grodkowska K., Gołębiewski M., Slósarz J., Grodkowski G., Kostusiak P., Sakowski T., Klopčič M., Puppel K. (2023). The Effect of Parity on the Quality of Colostrum of Holstein Dairy Cows in the Organic Production System. Animals.

[47] Morrill K.M., Conrad E., Lago A., Campbell J., Quigley J., Tyler H. (2012). Nationwide evaluation of quality and composition of colostrum on dairy farms in the United States. J. Dairy Sci..

[48] Cabral R., Chapman C., Aragona K., Clark E., Lunak M., Erickson P. (2016). Predicting Colostrum Quality from Performance in the Previous Lactation and Environmental Changes. J. Dairy Sci..

[49] Westhoff T.A., Overton T.R., Mann S. (2023). Epidemiology of Bovine Colostrum Production in New York Holstein Herds: Prepartum Nutrition and Metabolic Indicators. J. Dairy Sci..

[50] O’Hara A.E., Båge R., Emanuelson U., Holtenius K. (2019). Effects of Dry Period Length on Metabolic Status, Fertility, Udder Health, and Colostrum Production in 2 Cow Breeds. J. Dairy Sci..

